# Long-Term Career Earnings in Academia Might Offset the Opportunity Cost of Full-Time PhD and Postdoctoral Education for Physical Therapists Who Hold a Doctor of Physical Therapy Degree

**DOI:** 10.1093/ptj/pzad015

**Published:** 2023-02-20

**Authors:** Alexander J Garbin, Jennifer E Stevens-Lapsley, R Mark Gritz, Carole A Tucker, Michael J Bade

**Affiliations:** VA Eastern Colorado Geriatric Research, Education, and Clinical Center (GRECC), VA Eastern Colorado Health Care System, Aurora, Colorado, USA; Physical Therapy Program, Department of Physical Medicine and Rehabilitation, University of Colorado, Aurora, Colorado, USA; VA Eastern Colorado Geriatric Research, Education, and Clinical Center (GRECC), VA Eastern Colorado Health Care System, Aurora, Colorado, USA; Physical Therapy Program, Department of Physical Medicine and Rehabilitation, University of Colorado, Aurora, Colorado, USA; Adult and Child Consortium for Health Outcomes Research and Delivery Science, University of Colorado, Aurora, Colorado, USA; Division of Health Care Policy and Research, University of Colorado, Aurora, Colorado, USA; Nutrition, Metabolic & Rehabilitation Science Department, University of Texas Medical Branch - Galveston, Galveston, Texas, USA; VA Eastern Colorado Geriatric Research, Education, and Clinical Center (GRECC), VA Eastern Colorado Health Care System, Aurora, Colorado, USA; Physical Therapy Program, Department of Physical Medicine and Rehabilitation, University of Colorado, Aurora, Colorado, USA

**Keywords:** Cost–Benefit Analysis, Education: Faculty, Education: Professional, Physical Therapists

## Abstract

**Objective:**

Rigorously trained physical therapy researchers are essential for the generation of knowledge that guides the profession. However, there is a current and projected dearth of physical therapy researchers capable of sustaining research programs in part due to perceived financial barriers associated with pursuit of a doctor of philosophy (PhD) degree, with and without postdoctoral training, following doctor of physical therapy (DPT) degree completion. This study aimed to evaluate the financial impact of PhD and postdoctoral training, including opportunity cost, years to break even, and long-term earnings.

**Methods:**

Clinical and academic salaries were obtained via the 2016 APTA Median Income of Physical Therapist Summary Report and 2019 CAPTE Annual Accreditation Report. Salaries were adjusted to total compensation to account for benefits and compared over a 30-year period starting after DPT education. Total compensations were also adjusted to the present value, placing greater weight on early career earnings due to inflation and potential investments.

**Results:**

Relative to work as a clinical physical therapist, 4 years of PhD training result in an earnings deficit of $264,854 rising to $357,065 after 2 years of additional postdoctoral training. These deficits do not persist as evidenced by a clinical physical therapist career earning $449,372 less than a nonmajority scholarship academic career (DPT to PhD to academia pathway) and $698,704 less than a majority scholarship academic career (DPT to PhD to postdoctoral training to academia pathway) over a 30-year period. Greater long-term earnings for PhD careers persist when adjusting to present value.

**Conclusions:**

Although there is an initial opportunity cost of PhD and postdoctoral training represented by a relative earnings deficit, advanced research training results in greater long-term earnings.

**Impact:**

The findings of this study allow physical therapists interested in pursuing PhD and postdoctoral training to be better informed about the associated financial ramifications.

## Introduction

Physical therapy is an evidence-based profession that relies on the generation of new knowledge from a breadth of sources to guide our practice within ever-changing health and practice environments. Over the past decade, the critical relevance of rehabilitation research and the involvement of physical therapy researchers in diverse research domains (eg, regenerative medicine, pain sciences, qualitative research, health services research, and implementation sciences) has expanded. As the breadth of rehabilitation research has grown,^1^ the need for rigorously trained physical therapists who are prepared to develop sustained programs of research is even more critical. Furthermore, the physical therapist profession continues to mature, as evidenced by the American Physical Therapy Association (APTA)^2^ and Foundation for Physical Therapy Research^3^ each identifying the need for professional growth in research capacity.

Despite this emphasis on continued growth of the physical therapist knowledge base, there remains a relative shortage of rigorously trained [doctor of philosophy (PhD) or similar academic doctoral degrees] physical therapy researchers necessary to perform this work.^4^ This shortage of PhD-trained physical therapy researchers is particularly problematic within the US given the unprecedented growth in US-based physical therapist professional education programs, 264 programs in 2021 (compared with 230 in 2013) with 60 additional programs currently being developed.^5^ This large growth necessitates a similar growth in the number of PhD-trained physical therapy researchers and teaching faculty to retain high-quality evidence-informed training programs.^6^ In fact, the Commission on Accreditation in Physical Therapy Education (CAPTE) requires that 50% of core faculty in doctor of physical therapy (DPT) programs have academic doctoral degrees, a percentage which, on average, is not being achieved by existing DPT programs.^7^ In addition, according to a recent survey of PhD program directors from the Research-Intensive Programs in Physical Therapy consortium, 40% of physical therapist-related PhD programs are experiencing a decline in PhD applicants.^8^ The reported decline in PhD applicants in conjunction with the rapid growth in DPT education programs suggests that the percentage of core faculty with academic doctoral degrees will remain below the 50% mark required by CAPTE.

To address the shortage of high-quality PhD-trained physical therapist faculty, it is necessary to examine what impacts an individual’s decision to pursue a career in research. This is a complex issue influenced by a variety of factors including desire for full-time study and advanced training, intensive research, job and family responsibilities, and financial concerns.^9–11^ For DPT graduates, financial concern is a primary reason for the deferral of further post-graduate training.^8^ Given the average physical therapist student completes their undergraduate and DPT degrees with an education debt balance of $142,000,^12^ this concern is easy to understand. This debt likely places an increased emphasis on the reduced short-term earnings during 4 years of PhD and 2 years of postdoctoral training relative to a professional clinical position.

Though the potential for reduced short-term earnings while pursuing a PhD is noted as a perceived deterrent, understanding the longer-term positive financial consequences of a PhD deserves consideration. As such, the aim of this analysis was to examine the financial impact of a specific case scenario that includes pursuing full time PhD training directly after DPT graduation, with the purpose of entering academia. Specifically, we assessed (1) the initial relative loss in compensation of PhD training with or without additional postdoctoral training compared with working as a clinician (opportunity cost), (2) the number of years needed to work to break even after the opportunity cost, and (3) long-term (30-year) earnings. Accomplishing this aim will allow physical therapists interested in pursuing PhD and postdoctoral training to be better informed regarding the financial ramifications of this decision.

## Methodology

We examined differences in long-term earnings between PhD-trained academic physical therapist careers and non-PhD physical therapist careers in either academic or clinical settings. For the purposes of this study, “PhD” represents all terminal academic research degrees (eg, doctor of education, doctor of science) and scholarship refers to active engagement in research.

### Survey

To determine the average time spent at each academic rank and factors influencing academic promotion (eg, majority scholarship), a survey was designed by members of the study team and sent via an email to program directors (n = 242) from accredited DPT programs across the country between July 26, 2021 and August 14, 2021. Anonymous survey responses were collected electronically through Qualtrics. The survey was approved by the Colorado Multiple Institutional Review Board. Prior to the first question in the survey, respondents were provided with the study purpose and electronically acknowledged their informed consent.

### Estimating Salaries

Academic physical therapist faculty salaries were analyzed utilizing a dataset provided to the authors via special request that was collected from the 2019 CAPTE Annual Accreditation Report. This dataset contains 1738 faculty salaries (out of a possible 2733^13^ national full-time core faculty positions within DPT programs in 2019; 63.6% response rate) as well as data on highest degree earned (PhD or equivalent) and percent of effort in scholarship, allowing for further academic career stratification. Additional data from the CAPTE Annual Accreditation Report used to project academic physical therapist salaries include whether an individual is a program director (program director data excluded from analysis), whether an individual is a physical therapist (non-physical therapists excluded from analysis), annual salary (unadjusted for contract length), academic rank, and years working as faculty (1–3, 4–5, 6–10, 11–15, 16–20, 21–25, 26–30, 31–35, 36–40, 41–51). Data on the number of years working as faculty were combined with survey results detailing the average time at each academic rank to determine the median starting salaries for each rank within the CAPTE Annual Accreditation report. For example, the starting salary for an associate professor is represented by the median of associate professor salaries that had worked for 6 to 10 years as survey results indicated faculty are most often promoted to this rank after 6 years.

Clinical physical therapist salaries were analyzed via the 2016 APTA Median Income of Physical Therapist Summary Report.^14^ This report includes data on the median annual clinical physical therapist salaries grouped by number of years worked (1–4, 5–7, 8–10, 11–16, 17+). Salaries from the 2016 Summary Report were increased according to increases in the mean annual salary for physical therapists from 2016 to 2017 (0.99%), 2017 to 2018 (0.91%), and 2018 to 2019 (1.45%)^15^ to allow for comparisons to the 2019 CAPTE Annual Accreditation Report.

These datasets resulted in 4 distinct career groups: (1) clinical physical therapists, (2) academic non-PhD physical therapists, (3) academic PhD-trained physical therapists with less than 50% workload committed to scholarship, and (4) academic PhD-trained physical therapists with a majority of workload committed to scholarship. The academic non-PhD physical therapist group represents physical therapists that transition to an academic career and salary after initially working in the clinical setting. Both PhD-trained careers were assumed to have spent 4 years in full-time PhD training with a salary of $30,000, whereas the majority scholarship PhD career was assumed to have an additional 2 years of full-time postdoctoral training with a salary of $55,000. Salaries during PhD and postdoctoral training are based on the authors’ universities; however, we recognize these may differ on a university-by-university basis. Consequently, Table 1 outlines the impact of varying training salaries and time to complete training on opportunity cost, break-even points, and long-term earning potential. The assumption that the majority scholarship group pursues postdoctoral training is secondary to evidence that postdoctoral training enhances research productivity.^16^^,^^17^ Many individuals also have potential for additional sources of income during training (clinical work, scholarships, loan repayment awards); however, these opportunities vary considerably and were not included in the model but are discussed in Table 1.

**Table 1:** Table Detailing Assumptions Made in PhD Career Cost Analysis, Alternative Experiences, and Impact on the Outcomes of Opportunity Cost

**Assumption**	**Alternative**	**Impact to PhD Groups**
**PhD training begins directly after DPT degree completion**	Working as a clinician prior to pursuing PhD training	Increased relative deficit (opportunity cost) of training (compared with clinicians earning more later in their career) Later break-even point Reduced long-term earning
**30-year projection after completion of DPT degree**	Projection past 30 years	Positive long-term earning differential will grow with each additional year
**Years to completed PhD and postdoctoral training**	More years to complete training (eg, part time PhD, longer program)	Increased relative deficits (opportunity cost) during training period Later break-even point Reduced long-term earning
	Fewer years to complete training	Reduced relative deficits (opportunity cost) during training period Earlier break-even point Increased long-term earning
**Salary during PhD and postdoctoral training (based on authors’ universities)**	Lower salary	Increased relative deficits (opportunity cost) during training period Later break-even point Reduced long-term earning
	Higher Salary	Reduced relative deficits (opportunity cost) during training period Earlier break-even point Increased long-term earning
**No additional income opportunities during PhD training**	Greater earning during PhD through grants/scholarships	Reduced relative deficits (opportunity cost) during PhD training period Earlier break-even point Slightly greater long-term earning
	Greater earning during PhD through additional part-time positions (teaching, clinical work)	Reduced relative deficits (opportunity cost) during PhD training period Earlier break-even point Slightly greater long-term earning Potentially longer time to complete training (see above)

Academic and clinical careers were projected over a 30-year period starting when DPT education was completed and with salaries increasing to the corresponding starting salary for each rank for academic careers (assistant professor, associate professor, professor) and bins of number of years worked as a physical therapist for the clinical career (1–4, 5–7, 8–10, 11–16, 17+). Between periods of new ranks or time bins, salaries were increased by 1% annually as this approximates the annual increases seen in clinical physical therapist salaries from 2016 to 2019. A conservative 30-year projection was employed given the diverse age range of DPT graduates^18^ as well as the high prevalence of burnout within clinical physical therapist practice,^19^^,^^20^ which is suggestive of early retirement.^21^^,^^22^

### Adjusting to Total Compensation

Projected salaries for academic and clinical careers were adjusted to projected total compensation to account for differences in total benefits (eg, paid leave, supplemental pay, insurance, retirement savings). These adjustments were based on 2019 Bureau of Labor Statistics Employer Cost for Employee Compensation data.^23^ This data details the percent of total compensation that is due to salary and total benefits distinctly. For example, total compensation for a university employee is 66.6% salary and 33.4% total benefits, whereas total compensation for health care worker is 69.1% salary and 30.9% total benefits. Salaries were adjusted to total compensation by dividing salary by the percentage of salary to total compensation.

Differences in annual and 30-year cumulative total compensation were calculated between PhD academic physical therapist careers and non-PhD physical therapist careers (clinical and academic). Pursuing additional PhD and postdoctoral training is an economic investment as represented by reduced short-term earnings. As such, break-even points were calculated to determine the number of years necessary for cumulative total compensation of PhD academic physical therapist careers to equal that of the clinical and academic non-PhD physical therapist careers.

### Adjusting to Present Value

Annual total compensation differences were adjusted to present value. These adjustments are necessary as present value represents the concept that compensation today is more valuable than future identical compensation due to expected inflation and potential investments compounding over time, which is called the time value of money. The discount rate determines the amount future compensations are influenced by these factors. For this investigation, we have employed a discount rate of 3% for the present value analyses. A 3% discount rate was chosen as it is in line with recommendations from the Second Panel on Cost-Effectiveness in Health and Medicine, which consisted of members with expertise in cost-effectiveness analyses that met over the course of 3 years to establish recommendations by consensus.^24^ This panel recommended a 3% discount rate secondary to data on economic growth and inflation. Sensitivity analyses were performed with discount rates of 1% and 5% to account for potential differences in inflation and investments. For example, a 1% discount rate may be more applicable for some individuals with limited capacity to invest early career earnings secondary to student debt following DPT graduation. Conversely, a 5% discount rate may be applicable to individuals whose investments resulted in greater monetary returns (eg, early purchase of a home).

Present values were calculated by applying a discount rate (*r*) to annual differences in total compensation (*C*) over years (*n*) throughout the 30-year period.^25^


$$PV=\frac{C}{{\left(1+r\right)}^n}$$

To provide an example of this calculation, a $1,000 difference in annual total compensations 1 year in the future would have a present value of $970.87 due to the impact of inflation and inability to invest these funds the year prior.

For the analysis, net present values (NPVs) were then calculated as the sum of present values over the 30-year period. NPVs indicate the opportunity cost of PhD-trained academic physical therapist careers compared with non-PhD alternatives while accounting for inflation and the greater potential for investments (eg, stocks, bonds) to be made early in one’s career.

## Results

### Survey Results

The survey was completed by 75 DPT program directors representing the full range of university research categories (R1, R2, and Doctoral/Professional Universities). Based on the survey results (Suppl. Table), the average faculty member without a PhD initially works in clinical practice for greater than 7 years prior to assuming an assistant professor faculty position. Survey results also revealed that faculty spend an average of 6 years at the rank of assistant professor prior to promotion to associate professor and another 7 years at associate professor prior to promotion to professor. These time-to-promotion results were the same for both PhD groups, independent of percent effort into scholarship.

### Total Compensation

**Figure 1:**
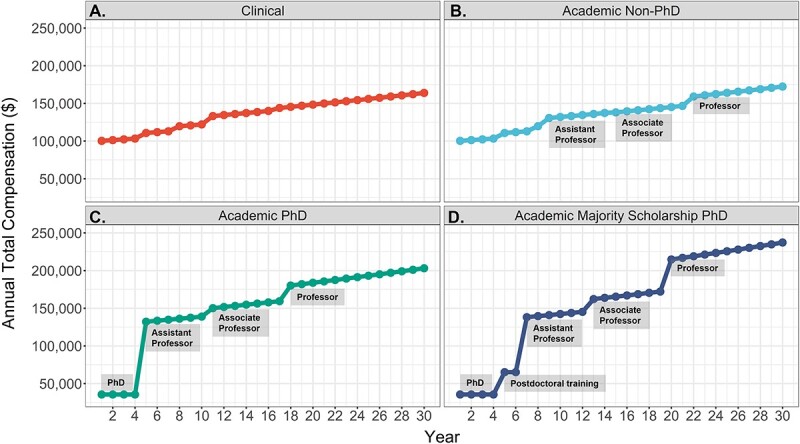
Annual total compensation of non-PhD and PhD physical therapist careers over a 30-year period. Careers examined include (A) clinical physical therapists, (B) academic non-PhD physical therapists, (C) academic PhD-trained physical therapists with less than 50% time commitment to scholarship, and (D) academic PhD-trained physical therapists with time spent in majority scholarship. The leftmost side of the gray boxes indicate the start of each training period and academic rank (B)–(D). The leftmost side of each graph represents DPT graduation.

**Figure 2:**
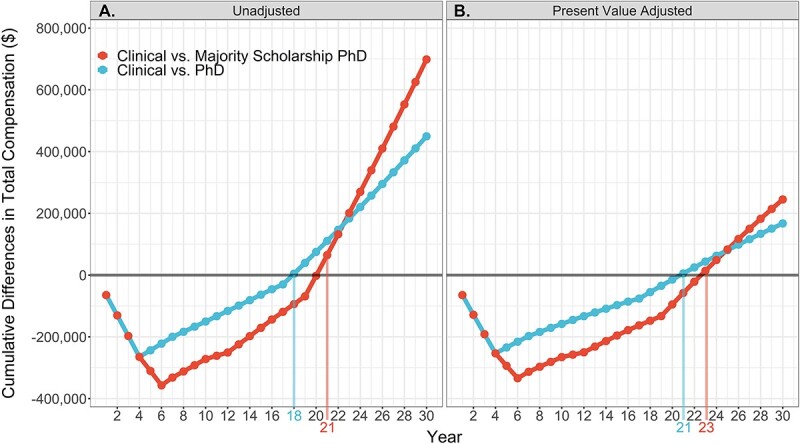
Differences in cumulative total compensation over time between a clinical physical therapist career and PhD physical therapist careers (A) unadjusted and (B) adjusted for present value with a 3% discount rate. Break-even points are indicated via a vertical line and number with colors matching each respective group comparison. The leftmost side of each graph represents DPT graduation.

Annual total compensations over time are illustrated for the career groups of (1) clinical physical therapists (Fig. 1A), (2) academic non-PhD physical therapists (Fig. 1B), (3) academic PhD-trained physical therapists with less than 50% time dedicated to scholarship (Fig. 1C), (4) academic PhD-trained physical therapists with time spent in majority scholarship (Fig. 1D). Total compensations are shown over a 30-year period following receipt of a DPT degree. Timelines of academic promotion, determined via program director survey, are also outlined. The results detail that PhD-trained careers experience reduced total compensation during PhD training (years 1–4) relative to their non-PhD counterparts. In addition, although total compensation increases for the majority scholarship PhD career during postdoctoral training (years 5–6), their compensation remains lower than the total compensation for the nonmajority scholarship PhD career (starting at the rank of assistant professor) and the non-PhD careers.

Although there is reduced short-term earnings for both PhD careers, their long-term earnings exceed that of the non-PhD careers (eg, clinical or academic non-PhD) following PhD and postdoctoral training. Furthermore, with each rank promotion, the difference in total compensation between PhD and non-PhD careers grows with larger increases in total compensation occurring for the PhD career involved in majority scholarship (Tab. 2).

**Table 2:** Median Annual Total Compensation and Compared Differences for Clinical Physical Therapists, Academic Non-PhD Physical Therapists, and Academic PhD-Trained Physical Therapists

	**Annual Total Compensation**				**Annual Total Compensation Differences** *^a^*			
**Year**	**Clinical Physical Therapist**	**Academic Non-PhD**	**PhD (Nonmajority Scholarship)**	**Majority Scholarship PhD**	**Clinical vs PhD (Nonmajority Scholarship)**	**Clinical vs Majority Scholarship PhD**	**Academic Non-PhD vs PhD (Nonmajority Scholarship)**	**Academic Non-PhD vs Majority Scholarship**
**1**	$100,245	$100,245	$35,545	$35,545	($64,700)	($64,700)	($64,700)	($64,700)
**2**	$101,247	$101,247	$35,545	$35,545	($65,702)	($65,702)	($65,702)	($65,702)
**3**	$102,260	$102,260	$35,545	$35,545	($66,715)	($66,715)	($66,715)	($66,715)
**4**	$103,282	$103,282	$35,545	$35,545	($67,737)	($67,737)	($67,737)	($67,737)
**5**	$110,718	$110,718	$132,132	$65,166	$21,414	($45,552)	$21,414	($45,552)
**6**	$111,825	$111,825	$133,453	$65,166	$21,628	($46,659)	$21,628	($46,659)
**7**	$112,943	$112,943	$134,788	$138,138	$21,845	$25,195	$21,845	$25,195
**8**	$119,695	$119,695	$136,136	$139,520	$16,441	$19,824	$16,441	$19,824
**9**	$120,892	$130,631	$137,497	$140,915	$16,605	$20,023	$6,867	$10,284
**10**	$122,101	$131,937	$138,872	$142,324	$16,771	$20,223	$6,935	$10,387
**11**	$133,161	$133,256	$150,150	$143,747	$16,989	$10,586	$16,894	$10,491
**12**	$134,492	$134,589	$151,652	$145,185	$17,159	$10,692	$17,063	$10,596
**13**	$135,837	$135,935	$153,168	$162,162	$17,331	$26,325	$17,233	$26,227
**14**	$137,196	$137,294	$154,700	$163,784	$17,504	$26,588	$17,406	$26,490
**15**	$138,568	$138,138	$156,247	$165,422	$17,679	$26,854	$18,109	$27,283
**16**	$139,953	$139,520	$157,809	$167,076	$17,856	$27,122	$18,290	$27,556
**17**	$144,008	$140,915	$159,387	$168,747	$15,379	$24,738	$18,473	$27,832
**18**	$145,448	$142,324	$180,180	$170,434	$34,732	$24,986	$37,856	$28,110
**19**	$146,903	$143,747	$181,982	$172,138	$35,079	$25,236	$38,235	$28,391
**20**	$148,372	$145,185	$183,802	$214,715	$35,430	$66,343	$38,617	$69,530
**21**	$149,856	$146,636	$185,640	$216,862	$35,784	$67,006	$39,003	$70,225
**22**	$151,354	$159,159	$187,496	$219,030	$36,142	$67,676	$28,337	$59,871
**23**	$152,868	$160,751	$189,371	$221,221	$36,504	$68,353	$28,620	$60,470
**24**	$154,396	$162,358	$191,265	$223,433	$36,869	$69,037	$28,907	$61,075
**25**	$155,940	$163,982	$193,178	$225,667	$37,237	$69,727	$29,196	$61,685
**26**	$157,500	$165,622	$195,109	$227,924	$37,610	$70,424	$29,488	$62,302
**27**	$159,075	$167,278	$197,060	$230,203	$37,986	$71,129	$29,783	$62,925
**28**	$160,665	$168,951	$199,031	$232,505	$38,366	$71,840	$30,080	$63,555
**29**	$162,272	$170,640	$201,021	$234,830	$38,749	$72,558	$30,381	$64,190
**30**	$163,895	$172,347	$203,032	$237,179	$39,137	$73,284	$30,685	$64,832
**SUM** *^b^*	$4,076,968	$4,153,408	$4,526,340	$4,775,672	$449,372	$698,704	$372,931	$622,264

^a^ Negative values (in parentheses) indicate greater compensation for the non-PhD careers, whereas positive values indicate greater compensation for the PhD careers.

^b^ Cumulative total compensations and differences over the 30-year period.

### Cumulative Differences and Break-Even Points

Additional insight into the raw differences in total compensation between the PhD careers and the clinical physical therapist careers revealed an initial earnings deficit for PhD careers followed by greater long-term earnings (Fig. 2A; Tab. 2). Specifically, over 4 years of PhD training, both PhD career paths receive $264,854 less total compensation than their non-PhD counterparts. For the majority scholarship PhD career, the relative loss in total compensation increases to $357,065 by the end of postdoctoral training. Despite the reduced short-term earnings, the increased total compensation after PhD training results in the nonmajority scholarship PhD career breaking even (no net loss or gain) at year 18 after DPT training (14 years after PhD training) relative to the clinical physical therapist career and year 19 (15 years after PhD training) relative to the non-PhD academic physical therapist career. The majority scholarship PhD career breaks even at year 21 after DPT training (15 years after postdoctoral training) relative to the clinical and non-PhD academic physical therapist careers. Both PhD careers also experience substantially greater long-term earnings over the 30-year period relative to the non-PhD careers (PhD vs clinical: +$449,372; PhD vs non-PhD academic: +$372,931; majority scholarship PhD vs clinical: +$698,704; majority scholarship PhD vs non-PhD academic: +$622,264).

### NPV

Adjusting differences to present value was more favorable to the non-PhD careers due to their greater early career total compensations (Fig. 2B; Tab. 3). However, both PhD careers still broke even relative to the non-PhD careers (PhD vs clinical: 21 years; PhD vs non-PhD academic: 22 years; majority scholarship PhD vs clinical: 23 years; majority scholarship PhD vs non-PhD academic: 23 years). Furthermore, a positive 30-year NPV was observed for the PhD careers relative to the non-PhD careers (PhD vs clinical: +$167,298; PhD vs non-PhD academic +$126,673; majority scholarship PhD vs clinical: +$245,167; majority scholarship vs non-PhD academic: +$204,543).

### Sensitivity Analyses

A more conservative discount rate of 1% resulted in PhD careers breaking even earlier (PhD vs clinical: 19 years; PhD vs non-PhD academic: 19 years; majority scholarship PhD vs clinical: 21 years; majority scholarship PhD vs non-PhD academic: 21 years) and having a larger positive 30-year NPV (PhD vs clinical: +$333,488; PhD vs non-PhD academic +$272,095; majority scholarship PhD vs clinical: +$510,109; majority scholarship vs non-PhD academic: +$448,715). With a less conservative 5% discount rate, both PhD careers still broke even (PhD vs clinical: 25 years; PhD vs non-PhD academic: 26 years; majority scholarship PhD vs clinical: 26 years; majority scholarship PhD vs non-PhD academic: 27 years) and had a positive 30-year NPV (PhD vs clinical: +$59,955; PhD vs non-PhD academic +$32,099; majority scholarship PhD vs clinical: +$79,601; majority scholarship vs non-PhD academic: +$51,745) (Tab. 3).

## Discussion

The APTA and Foundation for Physical Therapy Research have emphasized the need for increased research to continue the growth of the physical therapist profession. However, there is a shortage of high-quality PhD-trained physical therapy researchers and teaching faculty^4^ that will continue to rise with the decline in applicants at PhD programs related to physical therapist^8^ and concomitant growing number of DPT programs.^7^ Financial concerns are cited as a major barrier to physical therapists pursuing a PhD.^8^ As such, this study aimed to examine the opportunity cost, break-even points, and long-term earnings for a specific case scenario of DPT graduates that directly pursue a PhD with or without postdoctoral training relative to a career as a clinical physical therapist over a 30-year period.

Unsurprisingly, PhD-trained careers have reduced short-term earnings during their training period relative to a career as a clinical physical therapist or a clinical physical therapist that transitions into academia without PhD training. However, these reduced earnings do not persist. Both PhD-trained careers break even after 18 to 21 years and have substantially greater long-term earnings after 30 years than their non-PhD counterparts. Notably, the greatest gap in total compensation between PhD-trained physical therapists and clinical physical therapists occurs after PhD-trained physical therapists are promoted to the rank of Professor. As such, the difference in total compensation will continue to expand in favor of the PhD-trained careers if they work past the moderate 30-year time horizon utilized in this study.

Given the differences in early versus late career earnings, it is particularly important to adjust to present value to understand the impact of inflation and potential early career investments. Present value adjustments placed greater weight on the early career earnings, thus favoring clinical and academic non-PhD physical therapist careers. However, when adjusting to present value with the recommended discount rate of 3%, PhD careers still experienced greater 30-year cumulative earnings relative to non-PhD careers. The greater earnings persisted with a less conservative discount rate of 5% (representing a greater return on early career investments), albeit with later break-even points and reduced positive cumulative earnings differentials. These results demonstrate that the reduced potential for early career investments for PhD-trained careers relative to their non-PhD counterparts does not offset their greater late career earnings. It is important to note that the choice of discount rate strongly influences the resultant present value. Previous health care salary studies have used a variety of discount rates in their present value analyses.^26–31^ The Second Panel on Cost-Effectiveness in Health and Medicine created economic-based recommendations to reduce variability and improve comparability across studies. As such, we elected to use these recommendations for our choice of discount rate, 3% with additional sensitivity analyses. Future health care present value studies should carefully determine whether this discount rate approach is appropriate for their research question or if varying factors (eg, country of focus, long-term economic outlook) may necessitate a different discount rate.

The goal of these analyses was to create an accurate depiction of financial opportunity cost and long-term earnings for a specific case of DPT graduates that directly pursue full time PhD training relative to those who do not. However, several factors known to influence career earnings were unable to be represented in the analyses (Tab. 1). One important factor includes eligibility for programs that assist in repayment of educational debt, a large burden for many DPT graduates.^12^ The National Institute of Health Loan Repayment Program^32^ is one such program that targets health science researchers, typically with a PhD or in PhD training. Loan forgiveness programs for non-PhD physical therapist careers also exist; however, these programs often include strict eligibility criteria. For instance, one popular option, Public Service Loan Forgiveness,^33^ requires the applicant work for a government or not-for-profit agency for 10 years prior to being eligible. Another factor not represented in these analyses was the potential for scholarships during and after PhD and postdoctoral training. For instance, the Promotion of Doctoral Studies from the Foundation for Physical Therapy Research is a scholarship series often sought after by physical therapists undergoing PhD training.^34^ This scholarship series, National Institute of Health training grants^35^ and fellowships,^36^ and other possibilities for income (eg, part-time clinical work) can result in additional compensation that supports training and, in turn, partially offsets the reduced earnings experienced during this period.

In addition to factors outside of this study’s analyses that can impact finances, there are also factors within the analyses that may vary person-to-person and influence earnings. Time to complete PhD and postdoctoral training was set at 4 and 2 years, respectively; however, these timeframes can fluctuate depending on the program and individual.^37^ Though analyses for longer training periods were not performed, the authors anticipate that more years spent in PhD and/or postdoctoral training would still result in positive long-term earnings relative to a clinical physical therapist career given the early break-even points and large earnings differentials seen in the current analyses (Fig. 2). This study’s analyses also assumed PhD training would start immediately following DPT graduation. The decision to pursue a PhD following DPT graduation is increasingly common, but many individuals also elect to pursue a PhD after spending time in clinical practice. A longer clinical career prior to the start of PhD training would result in greater earnings deficits during the PhD and postdoctoral training phases secondary to a higher relative clinical physical therapist salary. Given the numerous factors capable of influencing the opportunity cost of pursuing a PhD, the authors of this study recommend these results be viewed as a starting point that can be adjusted based on individual opportunities and characteristics.

Although this study provided insight into the perceived financial barrier of pursuing PhD training for physical therapists contemplating this career pathway, numerous other factors may also influence one’s decision to pursue a PhD. Full-time study, fear of intensive research, and current job and family responsibilities have been cited as barriers to pursuing PhD education.^9–11^ In addition, future career aspirations (clinical vs teaching vs research) will play a major role in the decision to pursue a PhD. As such, the financial results of this study should be viewed together with these additional factors when weighing the decision to pursue a career as a PhD-trained physical therapist.

**Table 3:** Results of Sensitivity Analysis Detailing Present Value-Adjusted Differences*^a^*

	**1% Discount Rate**		**3% Discount Rate**		**5% Discount Rate**	
**Year**	**Clinical vs PhD (Nonmajority Scholarship) PV Differential**	**Clinical vs Majority Scholarship PhD** **PV Differential**	**Clinical vs PhD (Nonmajority Scholarship) PV Differential**	**Clinical vs Majority Scholarship PhD** **PV Differential**	**Clinical vs PhD (Nonmajority Scholarship) PV Differential**	**Clinical vs Majority Scholarship PhD** **PV Differential**
**1**	($64,700)	($64,700)	($64,700)	($64,700)	($64,700)	($64,700)
**2**	($65,052)	($65,052)	($63,788)	($63,788)	($62,573)	($62,573)
**3**	($65,400)	($65,400)	($62,885)	($62,885)	($60,512)	($60,512)
**4**	($65,745)	($65,745)	($61,989)	($61,989)	($58,514)	($58,514)
**5**	$20,579	($43,775)	$19,026	($40,472)	$17,617	($37,476)
**6**	$20,579	($44,395)	$18,657	($40,249)	$16,946	($36,559)
**7**	$20,579	$23,735	$18,294	$21,100	$16,301	$18,801
**8**	$15,335	$18,491	$13,368	$16,119	$11,684	$14,089
**9**	$15,335	$18,491	$13,108	$15,806	$11,239	$13,552
**10**	$15,335	$18,491	$12,854	$15,499	$10,811	$13,036
**11**	$15,380	$9,584	$12,642	$7,877	$10,430	$6,499
**12**	$15,380	$9,584	$12,396	$7,724	$10,033	$6,251
**13**	$15,380	$23,362	$12,155	$18,464	$9,650	$14,659
**14**	$15,380	$23,362	$11,919	$18,105	$9,283	$14,100
**15**	$15,380	$23,362	$11,688	$17,754	$8,929	$13,563
**16**	$15,380	$23,362	$11,461	$17,409	$8,589	$13,046
**17**	$13,116	$21,097	$9,584	$15,416	$7,045	$11,333
**18**	$29,327	$21,097	$21,013	$15,117	$15,153	$10,901
**19**	$29,327	$21,097	$20,605	$14,823	$14,576	$10,486
**20**	$29,327	$54,915	$20,205	$37,834	$14,021	$26,254
**21**	$29,327	$54,915	$19,813	$37,100	$13,487	$25,254
**22**	$29,327	$54,915	$19,428	$36,379	$12,973	$24,292
**23**	$29,327	$54,915	$19,051	$35,673	$12,479	$23,367
**24**	$29,327	$54,915	$18,681	$34,980	$12,003	$22,476
**25**	$29,327	$54,915	$18,318	$34,301	$11,546	$21,620
**26**	$29,327	$54,915	$17,963	$33,635	$11,106	$20,796
**27**	$29,327	$54,915	$17,614	$32,982	$10,683	$20,004
**28**	$29,327	$54,915	$17,272	$32,342	$10,276	$19,242
**29**	$29,327	$54,915	$16,936	$31,714	$9,885	$18,509
**30**	$29,327	$54,915	$16,608	$31,098	$9,508	$17,804
**NPV**	**$333,488**	**$510,109**	**$167,298**	**$245,167**	**$59,955**	**$79,601**

^a^ Results of sensitivity analysis detailing present value-adjusted differences, rounded to the nearest whole dollar, with a discount rate of 1% (most conservative), 3%, and 5% (least conservative) for clinical physical therapist career vs PhD career pathways. Negative values (in parentheses) indicate greater compensation for the non-PhD careers, whereas positive values indicate greater compensation for the PhD careers. NPV = net present value; PV = present value

### Limitations

There were limitations within this study, most notably the use of salary data from 2019, the most recent available academic careers salary data, and 2016 (adjusted to 2019) for the clinical career. Although the utilization of past salary data is a limitation, the authors believe these data are appropriate to use, as salary data collected during the COVID-19 pandemic may not reflect typical normative salaries. This is particularly true for clinical physical therapists, many of whom have experienced reductions in hours and income.^38^ An additional limitation is the use of training stipends and salaries taken at a single point in time. Consequently, it is important to note that these are best estimates of future salaries for DPT students considering pursuing PhD training; however, it is possible that these values may differ. Although not a limitation, there were factors within the analyses (time from completion of DPT education to the start of PhD education, time to complete additional PhD/postdoctoral training, varying stipend amounts during training, and differing lengths of academic and clinical careers) that may vary and influence outcomes. A sensitivity analysis for these factors was outside of the scope of this study. As such, Figures 1 and 2 and Table 1 are presented within this manuscript, in part, to allow readers to visualize and understand how changes in these factors might impact opportunity cost, break-even points, and long-term earnings differentials. However, there is a need for future work to examine how these factors vary within the physical therapist profession and assess their impact on finances.

## Conclusion

Although financial concerns are often cited cost as a barrier to enter PhD training, our analyses demonstrated that the long-term earnings are greater for those that pursue a PhD in lieu of short-term earnings. These findings will allow physical therapists to be better informed when considering the option of pursuing PhD training.

## Supplementary Material

Supplementary_Table_tsr_pzad015

## Data Availability

The authors confirm that the data supporting the findings of this study are available within the article.
