# Supplementary material for: Long-Term Career Earnings in Academia Might Offset the Opportunity Cost of Full-Time PhD and Postdoctoral Education for Physical Therapists Who Hold a Doctor of Physical Therapy Degree
Source: Phys Ther. 2023 Feb 20;103(4):pzad015. doi: 10.1093/ptj/pzad015 (PMC10152085; doi:10.1093/ptj/pzad015)
Supplement: Supplementary_Table_tsr_pzad015 [file supplementary_table_tsr_pzad015.pdf]

Supplementary Table. Survey responses from program directors of accredited DPT programs.

Questions<br>Total responses (n=75)		Response, Mean (SD)
**What is your University's Carnegie Classification? Count (%)**		
	R1 - Very high research activity	19 (25.3%)
	R2 - High research activity	11 (14.7%)
	D/PU - Doctoral or Professional University	38 (50.7%)
	Did not know	7 (9.3%)
**Currently, how many core full-time faculty are in your DPT program?**		12.10 (5.40)
**How many current full time core faculty have an advanced, post-professional degree? (n=70)**		6.80 (2.50)
**For your current faculty with post-professional degrees, approximately how many had one year or more of post-doc experience prior to their initial academic position? (n=73)**		2.12 (2.02)
**For your current faculty with post-professional degrees, approximately how many had NO post-doc experience prior to their initial academic position? (n=68)**		5.22 (2.40)
**For faculty members with PT degrees (no post-professional degree) with greater than or equal to 0.5 FTE faculty position:<br>How many years of clinical experience do most have prior to assuming their initial faculty position?**		6.50 (1.40)
**For individuals in a faculty "Teaching/Instruction" position (e.g. less than 50% research responsibilities).<br>What is the average number of years at the Instructor level prior to promotion to Assistant Professor? (n=33)**		2.91 (1.33)
**For individuals in a faculty "Teaching/Instruction" position (e.g. less than 50% research responsibilities).<br>What is the average number of years at the Assistant Professor level prior to promotion to Associate Professor? (n=74)**		5.99 (1.54)
**For individuals in a faculty "Teaching/Instruction" position (e.g. less than 50% research responsibilities or is NOT on a tenure track).<br>What is the average number of years at the Associate Professor level prior to promotion to Full Professor? (n=61)**		6.84 (2.31)
**For individuals in a faculty "RESEARCH" position (e.g. greater than 50% research responsibilities).<br>What is the average number of years at the Instructor level prior to promotion to Assistant Professor? (n=10)**		2.40 (0.70)
**For individuals in a faculty "RESEARCH" position (e.g. greater than 50% research responsibilities).<br>What is the average number of years at the Assistant Professor level prior to promotion to Associate Professor? (n=44)**		6.16 (1.27)
**For individuals in a faculty "RESEARCH" position (e.g. greater than 50% research responsibilities).<br>What is the average number of years at the Associate Professor level prior to promotion to Full Professor? (n=37)**		6.95 (2.25)
